# Prognostic value of the early D-dimer/fibrinogen ratio in critically ill patients requiring vascular surgery: a retrospective study

**DOI:** 10.1186/s12871-026-03924-7

**Published:** 2026-05-22

**Authors:** Zhao Liu, Eerdunbilige, Bin Liu, Hai Feng

**Affiliations:** 1 Department of Vascular Surgery, Beijing Friendship Hospital, Capital Medical University, Beijing, 100050 China; 2 Department of Vascular Surgery, International Mongolian Hospital of Inner Mongolian, Hohhot, 010020 China

**Keywords:** Clinical decision making, Critical care, D-dimer, Fibrinogen, Prognostic factors, Vascular surgery

## Abstract

**Background:**

Patients requiring vascular surgery who are managed in intensive care units (ICUs) commonly have perioperative coagulation abnormalities. These anomalies, which can be accessed via measurement of D-dimer and fibrinogen levels, influence patient outcomes. We aimed to evaluate the prognostic value and clinical significance of the D-dimer/fibrinogen ratio (DFR) measured within 6 h of ICU admission in critically ill vascular patients.

**Methods:**

This retrospective study included data from 297 patients (70.4% men; average age, 57.0 years) admitted to the ICUs of Beijing Friendship Hospital with vascular diseases between 2021 and 2025. We constructed multivariate regression models, via backward stepwise elimination, to identify independent predictors of in-hospital mortality and organ dysfunction.

**Results:**

Non-survivors (12.1%) had a significantly higher DFR than survivors (3.5 versus 1.1, *P* < 0.01). The multivariate regression model identified DFR as an independent predictor of in-hospital mortality (odds ratio = 1.37, 95% confidence interval: 1.05–1.80, *P* < 0.05). Higher DFR also significantly correlated with lower platelet counts (β=−6.83, *P* < 0.05) and higher N-terminal pro-B-type natriuretic peptide levels (β = 969, *P* < 0.01) in the multivariate model. Overall, the DFR-based predictive model demonstrated high precision (area under the curve of 0.861 for the overall cohort, 0.942 for patients with aortic dissection/aneurysm, and 0.927 for those with Thrombotic diseases).

**Conclusion:**

Our findings suggest that, given its high predictive accuracy and robustness across different vascular pathologies, early DFR, as assessed via routinely available laboratory parameters, has the potential to guide prognosis assessment and risk stratification in vascular ICU settings.

## Background

Critically ill surgery patients commonly have perioperative coagulation abnormalities, which represent a major clinical challenge because they can increase bleeding and thrombotic risks, complicate operative timing, and influence postoperative organ dysfunction [1, 2]. This issue is particularly relevant in vascular surgery, in which patients frequently present with preoperative acute vascular injury, tissue ischemia, and systemic inflammatory activation [3]. Such conditions can trigger marked activation of coagulation and fibrinolysis even before definitive intervention, making the perioperative period a high-risk window for hemostatic derangements and adverse outcomes. Validated approaches for risk stratification of critically ill patients requiring vascular surgery could improve clinical decision making, thereby improving patient care and making the best use of limited critical care resources [4].

Current perioperative assessment of coagulation relies on clinical bleeding/thrombosis history, standard laboratory tests (e.g., platelet count, prothrombin time, activated partial thromboplastin time, and fibrinogen), and fibrinolysis-related markers. Among these, D-dimer reflects cross-linked fibrin degradation and is widely used as an indicator of fibrin turnover. However, in critically ill perioperative patients, D-dimer elevation is often non-specifically driven by numerous factors, including intravascular thrombosis, fibrinolysis, surgery-related tissue injury, and systemic inflammation [5]. The concentration of fibrinogen, both an essential clotting substrate and an acute-phase reactant, can vary with increased consumption or inflammation-driven synthesis [6]. As such, D-dimer or fibrinogen level in isolation may not reliably distinguish predominant coagulation activation from inflammatory upregulation in complex perioperative vascular patients [2].

Instead, the D-dimer/fibrinogen ratio (DFR) has been proposed as an “integrated hemostasis” index that contextualizes fibrin degradation relative to available fibrinogen reserve [7–11]. In individuals with vascular pathologies, D-dimer and fibrinogen levels often move in opposite directions, depending on the interplay of inflammation-driven synthesis, coagulation, and consumption [11, 12], and DFR may offer prognostic information for acute vascular conditions [13, 14]. With its ability to report complex, dynamic changes in coagulation status, DFR is particularly useful in vascular emergencies, in which endothelial disruption, tissue-factor exposure, and systemic inflammatory activation can accelerate thrombin generation and fibrin deposition while simultaneously activating fibrinolysis [10–12].

It remains incompletely understood how DFR relates to outcomes for perioperative vascular surgery patients managed in the intensive care unit (ICU). Preoperative disease biology (e.g., aortic pathology or thrombotic disease) and perioperative interventions (e.g., surgery/endovascular repair, transfusion, or anticoagulation) in these patients can rapidly alter D-dimer and fibrinogen dynamics [15–17]. Acute aortic dissection is associated with tissue factor-mediated coagulation activation, platelet activation, and false-lumen thrombosis [18]. Thrombotic diseases (e.g., deep vein thrombosis and pulmonary embolism) are commonly characterized by elevated D-dimer levels owing to active clot formation and breakdown; D-dimer is used in diagnostic and prognostic pathways for Thrombotic diseases, although it is not fully specific in critical illness [4, 19, 20].

In a retrospective observational study, we aimed to evaluate the prognostic value of early DFR for the primary outcomes of in-hospital mortality, length of stay, ICU-free days, and mechanical ventilation-free time, as well as clinically relevant secondary outcomes, in critically ill patients with vascular pathologies including aortic dissection/aneurysm and Thrombotic diseases.

## Methods

### Study design and population

**Fig. 1:**
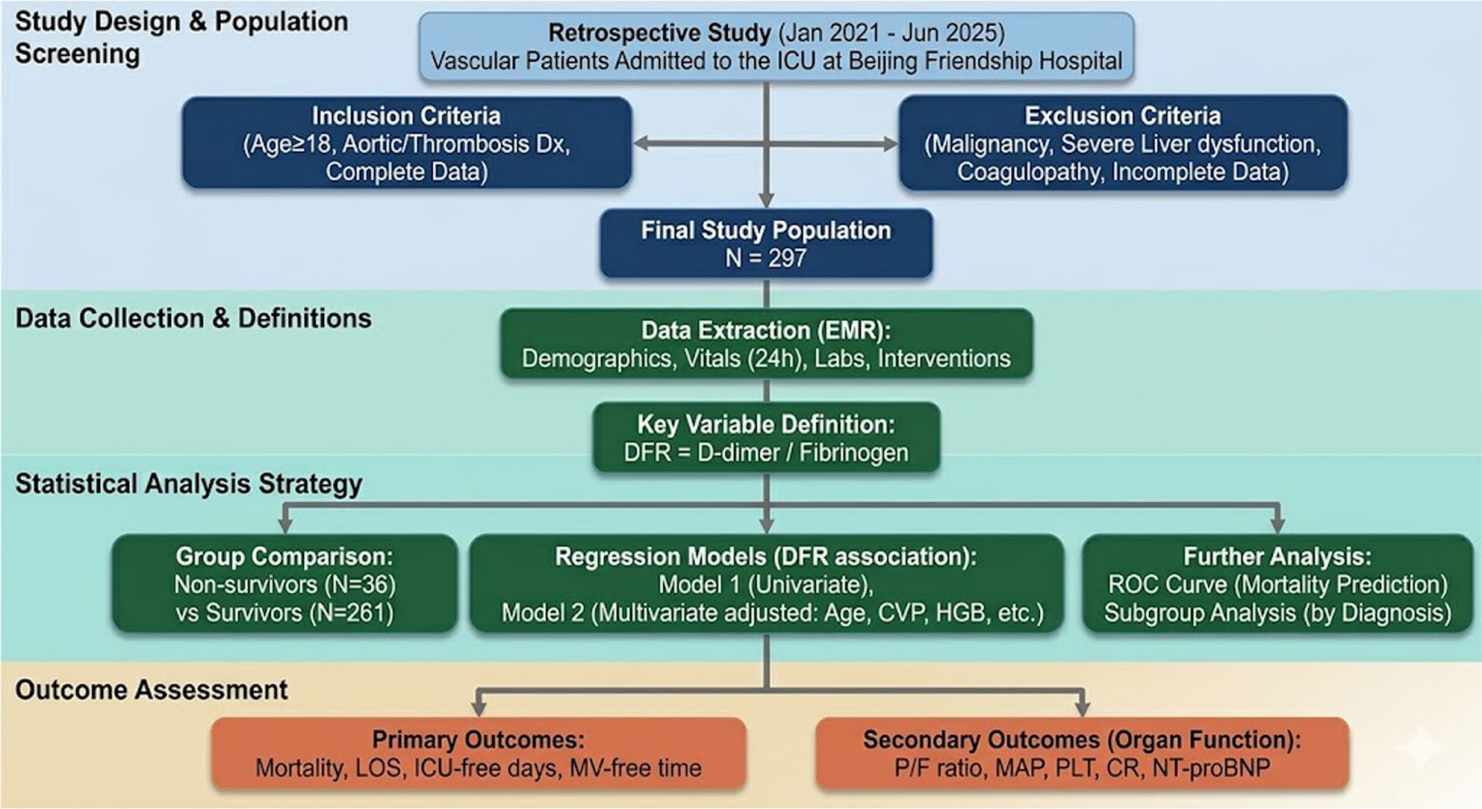
Study flowchart. The flowchart depicts the study population and design, and provides details of data collection and statistical analysis. Dx, diagnosis; EMR, electronic medical record; CVP, central venous pressure; HGB, hemoglobin; ROC, receiver operating characteristic; LOS, length of stay; MV, mechanical ventilation; P/F, ratio of arterial oxygen partial pressure to fractional inspired oxygen; MAP, mean arterial pressure; PLT, platelet count; CR, creatinine; DX, Diagnosis; EMR, Electronic Medical Record

This was a retrospective cohort study conducted to investigate the association between DFR and clinical outcomes in patients in the ICU with vascular diseases. The study population included 297 perioperative patients with vascular diseases admitted to the ICU of Beijing Friendship Hospital between Jan 1st, 2021, and June 30th, 2025. Eligibility criteria were: (1) age ≥ 18 years; (2) confirmed diagnosis of either aortic dissection/aneurysm or Thrombotic diseases (e.g., deep vein thrombosis or pulmonary embolism) via imaging examinations (i.e., computed tomography angiography, magnetic resonance angiography, or ultrasound) and laboratory tests; and (3) availability of complete clinical data, including baseline demographics, vital signs, laboratory results, and follow-up information until hospital discharge or death. Patients who met the eligibility criteria, but whose preoperative assessments revealed that surgery would pose an unacceptable risk were instead managed with conservative treatment (*n* = 25). Patients with active malignancy, severe liver dysfunction (i.e., Child-Pugh class C), coagulation disorders unrelated to vascular diseases, or incomplete data were excluded. A flowchart outlining the study population and detailing the data collection and statistical analysis procedures is presented in Fig. 1.

### Ethical approval

The study protocol was approved by the Institutional Review Board of Beijing Friendship Hospital (Approval No.: 2020-P2-073-02) and complied with the principles of the Declaration of Helsinki and its amendments. Informed consent was waived owing to the retrospective nature of the study.

### Data collection

Data were extracted from the hospital’s electronic medical record system by four independent researchers, with discrepancies resolved through discussion with a fifth senior clinician. The collected variables included the following: (1) baseline demographics (i.e., age and sex), clinical interventions and diagnoses (i.e., mechanical ventilation time, history of surgery during hospitalization, and primary diagnosis [dissection/aneurysm or Thrombotic diseases]); (2) vital signs, including heart rate, respiratory rate, maximum body temperature, central venous pressure, and mean arterial pressure, all measured within 24 h of ICU admission; and (3) laboratory markers, including lactate, white blood cell count, hemoglobin, blood urea nitrogen, cardiac troponin I, fibrinogen, D-dimer, platelet count, creatinine, total bilirubin, and N-terminal pro-B-type natriuretic peptide (NT-proBNP). All laboratory tests were performed using standard methods in the hospital’s clinical laboratory. DFR was calculated as follows: DFR = D-dimer/fibrinogen. Early DFR was defined as the first DFR in ICU.

### Outcomes

We measured the predictive value of DFR for primary and secondary clinical outcomes. Primary outcomes included in-hospital mortality, length of stay, ICU-free days, and mechanical ventilation-free time. Secondary outcomes were measures of organ system function based on Sequential Organ Failure Assessment scores, including the ratio of arterial oxygen partial pressure to fractional inspired oxygen, mean arterial pressure, platelet count, total bilirubin, creatinine, and NT-proBNP.

### Statistical analysis

Statistical analyses were performed using SPSS 26.0 (IBM Corp., Armonk, NY, USA) and R 4.2.1 (R Foundation for Statistical Computing, Vienna, Austria). Continuous variables with non-normal distribution (assessed via Shapiro–Wilk test) were presented as median (interquartile range), and comparisons between the survivors and non-survivors were conducted using the Mann–Whitney U test. Categorical variables were expressed as counts (percentages), and group comparisons were performed using the χ² test or Fisher’s exact test (when expected frequency < 5). Continuous variables with highly skewed distributions, such as NT-proBNP, were not log-transformed prior to regression analysis. We accounted for this skewing by utilizing a generalized estimating equation model with robust standard errors, which relaxes the strict distributional assumptions required by traditional linear regression.

To evaluate the association between DFR and outcomes, two regression models were constructed.

Model 1 is a univariate logistic regression (for binary outcome: in-hospital mortality) or linear regression (for continuous outcomes: length of stay, ICU-free days, mechanical ventilation-free time, ratio of arterial oxygen partial pressure to fractional inspired oxygen, mean arterial pressure, platelet count, total bilirubin, creatinine, and NT-proBNP) with DFR as the independent variable.

Model 2 is a multivariate model developed using a backward stepwise elimination procedure. Variables considered for entry included those with clinical relevance or for which *P* < 0.10 in the univariate model; we excluded variables for which *P* > 0.10. The final model was optimized for parsimony and clinical utility, adjusting for age, gender, central venous pressure, hemoglobin, lactate, lymphocyte count, neutrophil count, perfusion index, and white blood cell count to minimize confounding effects.

The predictive performance for in-hospital mortality was assessed by calculating the AUC for receiver operating characteristic curves. In the prediction equation, male = 1 and female = 0 for the sex variable. Subgroup analyses were further conducted by primary diagnosis (i.e., dissection/aneurysm versus Thrombotic diseases) to explore the consistency of DFR’s association with outcomes across subgroups. A two-sided P-value < 0.05 was considered statistically significant.

## Results

### Baseline characteristics and differences between the survivors and non-survivors

A total of 297 patients were enrolled in this study (Fig. 1), of whom 36 (12.1%) died (non-survivors) and 261 (87.9%) survived (survivors; Table 1). The median age of the overall population was 57.0 years, and there was no significant difference in age between the non-survivors and survivors. Gender distribution was also similar between the two groups (*P* > 0.10), with men accounting for 70.4% of the total population.

**Table 1:** Demographic characteristics of the study population

	Total	Survivors	Non-survivors	*P*-value
Patients	297	261	36	
Age (years)	57.0 (45.0–69.0)	57.0 (45.0–67.0)	60.0 (43.0–78.0)	> 0.10
Sex				> 0.10
Female	88 (29.6%)	77 (29.5%)	11 (30.6%)	
Male	209 (70.4%)	184 (70.5%)	25 (69.4%)	
Mechanical ventilation				**< 0.05**
No	52 (17.5%)	50 (19.2%)	2 (5.6%)	
Yes	245 (82.5%)	211 (80.8%)	34 (94.4%)	
Diagnosis				> 0.10
Dissection/ Aneurysm	164 (55.2%)	146 (55.9%)	18 (50%)	
Thrombotic diseases	133 (44.8%)	115 (44.1%)	18 (50%)	
Surgery				> 0.05
No	25 (8.4%)	19 (7.3%)	6 (16.7%)	
Yes	272 (91.6%)	242 (92.7%)	30 (83.3%)	
Heart rate (bpm)	114 (99–128)	112 (98–125)	130 (116–142)	**< 0.001**
Respiratory rate (breaths/minute)	23.0 (18.0–28.0)	23.0 (17.0–27.0)	28.0 (20.0–30.0)	**< 0.05**
Maximum body temperature (°C)	37.7 (37.0–38.3)	37.7 (37.1–38.3)	37.5 (36.8–38.5)	> 0.10
CVP (mm Hg)	8.0 (7.0–10.0)	8.0 (7.0–10.0)	9.0 (6.5–12.5)	> 0.05
Lactate (mmol/L)	2.9 (1.5–7.8)	2.6 (1.4–6.6)	6.6 (3.1–15.9)	**< 0.001**
White blood cell count (×10^9^/L)	11.6 (8.6–15.9)	11.0 (8.3–15.2)	17.1 (12.1–20.8)	**< 0.001**
Hemoglobin (g/L)	113 (96–126)	113 (97–125)	105 (91–130)	> 0.10
Blood urea nitrogen (mg/dL)	6.4 (4.7–10.0)	5.9 (4.5–9.1)	12.8 (8.2–18.2)	**< 0.001**
Cardiac troponin I (ng/mL)	0.1 (0.0–6.1)	0.1 (0.0–5.9)	0.8 (0.1–7.4)	**0.001**
Fibrinogen (g/dL)	2.9 (2.0–3.8)	2.9 (2.1–3.8)	2.4 (1.6–3.6)	**< 0.05**
D-dimer (mg/L)	3.6 (1.6–8.1)	3.5 (1.5–7.8)	7.4 (3.7–15.3)	**< 0.001**
DFR	1.2 (0.5–3.0)	1.1 (0.5–2.5)	3.5 (1.3–5.5)	**< 0.01**

*bpm* beats/minute

Bold: The effect size is statistically significant

Regarding clinical interventions and diagnoses, mechanical ventilation occurred significantly more frequently among non-survivors (94.4% versus 80.8%, *P* < 0.05), and the proportion of patients who underwent surgery was slightly lower among non-survivors (83.3% versus 92.7%), but this did not reach statistical significance. The diagnosis type (i.e., dissection/aneurysm versus thrombosis-related disease) did not differ between the survivors and non-survivors.

We observed significant inter-group disparities in vital signs and laboratory markers. Namely, the non-survivors had a higher median heart rate (130 versus 112 bpm, *P* < 0.001) and respiratory rate (28.0 versus 23.0 breaths per minute, *P* < 0.05). The non-survivors had significantly higher lactate levels, white blood cell counts, blood urea nitrogen levels, cardiac troponin I levels, D-dimer levels, and D-dimer/fibrinogen ratio, but lower fibrinogen levels. No significant differences were observed in maximum temperature, central venous pressure, or hemoglobin levels.

### Organ function and clinical outcomes

In terms of clinical outcomes, the non-survivors had a significantly shorter length of stay (5.1 versus 15.0 days, *P* < 0.05), fewer ICU-free days (0.8 versus 9.9 days, *P* < 0.05), and less mechanical ventilation-free time (1.3 versus 13.1 h, *P* < 0.001) (Table 2). Regarding readouts of organ system function, the non-survivors had a lower mean arterial pressure, lower arterial oxygen partial pressure-to-fractional inspired oxygen ratio, lower platelet count, higher creatinine levels, and significantly higher NT-proBNP levels. Bilirubin levels did not differ between groups (*P* > 0.10).

**Table 2:** Organ function parameters and clinical outcomes of the study population

	Total	Survivors	Non-survivors	*P*-value
Patients	297	261	36	
Length of stay (days)	13.9 (7.9–22.0)	15.0 (9.0–22.6)	5.1 (2.0–16.2)	**< 0.05**
ICU-free days	8.9 (4.2–16.8)	9.9 (4.9–17.0)	0.8 (0.3–7.3)	**< 0.05**
MV-free time (hours)	12.0 (6.8–20.5)	13.1 (8.3–21.0)	1.3 (0.8–12.3)	**< 0** **.001**
Mean arterial pressure (mmHg)	67.0 (60.0–75.0)	67.0 (60.5–75.0)	61.0 (52.0–70.0)	**< 0.01**
P/F	334.8 (242.0–432.5)	342.5 (251–439.1)	249.1 (150.3–351.9)	**< 0.05**
Platelets (×10^9^/L)	161 (110–208)	166 (114–210)	117 (89–173)	**< 0.01**
Creatinine (µmol/L)	86.5 (63.0–133.2)	81.5 (62.8–116.0)	158.0 (101.0–279.0)	**< 0.001**
Bilirubin (µmol/L)	16.6 (12.1–25.2)	16.5 (12.1–24.6)	18.0 (11.9–35.2)	> 0.10
NT-proBNP (pg/mL)	568 (155–2,826.5)	432 (140–2,252)	5,764 (484–13,224)	**< 0.001**

*MV* Mechanical ventilation, *P/F* ratio of arterial oxygen partial pressure to fractional inspired oxygen

Bold: The effect size is statistically significant

### Association between DFR and outcomes

In the univariate analysis (Model 1), DFR was significantly associated with increased in-hospital mortality (odds ratio [OR] = 1.71, 95% confidence interval [CI]: 1.36–2.15), decreased mean arterial pressure (β=−1.49 mmHg, 95% CI: −2.49 to − 0.49 mmHg), decreased platelet counts (β=−11.92 × 10⁹/L, 95% CI: −19.17 to − 4.66 × 10⁹/L), increased creatinine levels (β = 24.90 µmol/L, 95% CI: 8.99 to 40.80 µmol/L), and increased NT-proBNP levels (β = 1,134 pg/mL, 95% CI: 479 to 1,189 pg/mL) (Table 3). After adjusting for confounders (Model 2), DFR remained significantly associated with in-hospital mortality (odds ratio = 1.37, 95% CI: 1.05 to 1.80), lower platelet counts (95% CI: −6.83 to 0.08 × 10⁹/L, *P* < 0.05), and increased NT-proBNP (β = 969 pg/mL, 95% CI: 332 to 1,606 pg/mL). No significant associations were found between DFR and length of stay, ICU-free days, mechanical ventilation-free time, arterial oxygen partial pressure-to-fractional inspired oxygen ratio, or total bilirubin in either model.

**Table 3:** DFR predictive performance for primary and secondary outcomes

	Model 1	*P*-value	Model 2	*P*-value
In-hospital mortality	1.71 (1.36, 2.15)	< 0.001	1.37 (1.05, 1.80)	< 0.05
Length of stay (days)	1.17 (− 1.21, 3.55)	0.34	−1.27 (− 3.60, 1.06)	> 0.10
ICU-free days	0.85 (− 1.27, 2.96)	0.43	−0.82 (− 2.93, 1.29)	> 0.10
MV-free time (days)	1.00 (− 1.26, 3.27)	0.38	−0.95 (− 3.18, 1.27)	> 0.10
P/F	−4.09 (− 22.79, 14.62)	0.67	2.35 (− 16.13, 20.82)	> 0.10
MAP (mmHg)	**−1.49 (− 2.49**,** − 0.49)**	**0.004**	−0.11 (− 0.97, 0.75)	> 0.10
Platelets (×10^9^/L)	**−11.92 (− 19.17**,** − 4.66)**	**0.001**	**−6.83 (− 13.58**,** − 0.08)**	**< 0.05**
Total bilirubin (µmol/L)	−1.20 (− 4.40, 1.99)	0.46	−2.79 (− 6.06, 0.47)	> 0.05
Creatinine (µmol/L)	**24.90 (8.99**,** 40.80)**	**0.002**	12.25 (− 3.56, 28.05)	> 0.10
NT-proBNP (pg/mL)	**1**,**134 (479**,** 1189)**	**0.001**	**969 (332**,** 1**,**606)**	**< 0** **.01**

Model 1 is a univariate model. Model 2 is a multivariate model adjusted for age, central venous pressure, sex, hemoglobin, lactate, lymphocyte count, neutrophil count, perfusion index, and white blood cell count

*MAP* Mean arterial pressure, *MV* Mechanical ventilation, *P/F* ratio of arterial oxygen partial pressure to fractional inspired oxygen

Bold: The effect size is statistically significant

**Fig. 2:**
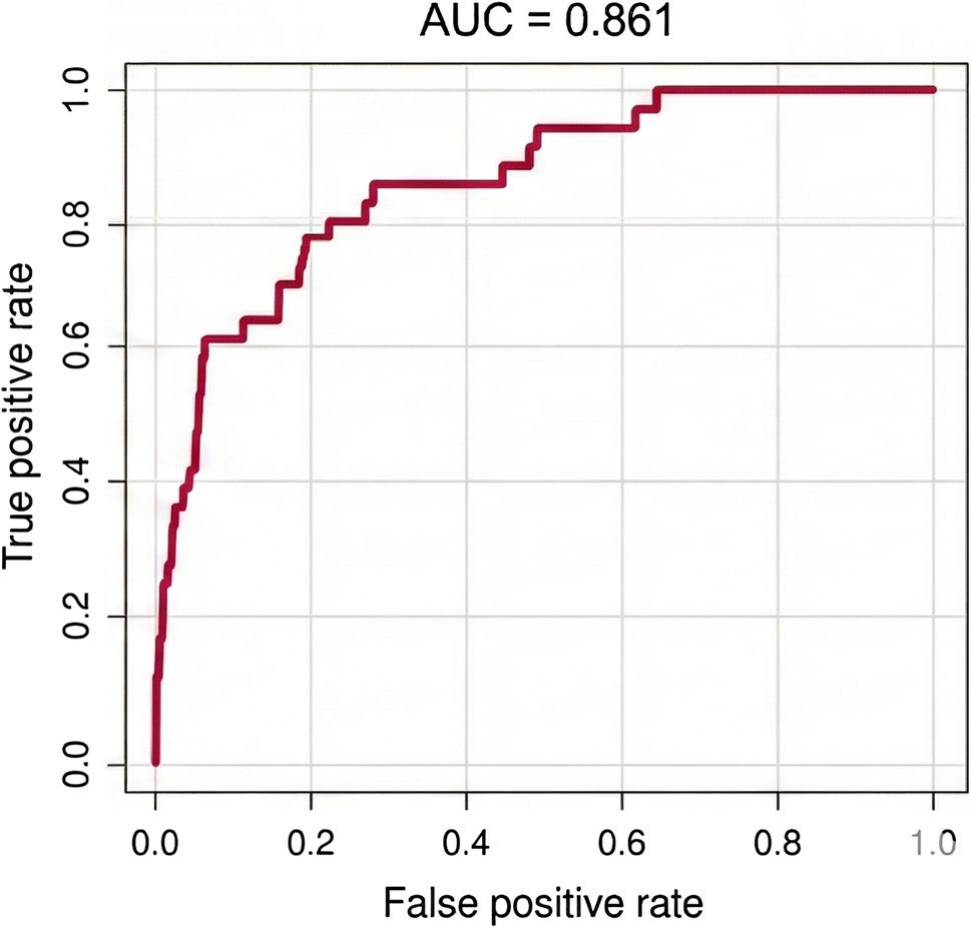
Early D-dimer/fibrinogen ratio has high predictive value for in-hospital mortality of vascular surgery patients managed in the intensive care unit. Receiver operating characteristic curve for in-hospital mortality in the multivariable model. The curve represents the equation: In-hospital mortality = − 2.399 + 0.025×age + 0.031×central venous pressure + 0.289×DFR − 0.119×(sex = 1) − 0.002×hemoglobin level + 0.011×lactate level − 0.010×lymphocyte count + 0.990×neutrophil count − 1.619×perfusion index − 0.176×((white blood cell count/100)⁻¹), where sex = 1 for male patients. DFR, D-dimer/fibrinogen ratio

The receiver operating characteristic curve for in-hospital mortality in the multivariable model had an area under the curve (AUC) of 0.861, indicating that the model displayed good predictive performance (Fig. 2). The predictive equation was as follows:

In-hospital mortality = − 2.399 + 0.025×age + 0.031×central venous pressure + 0.289×DFR − 0.119×(sex = 1) − 0.002×hemoglobin level + 0.011×lactate level − 0.010×lymphocyte count + 0.990×neutrophil count − 1.619×perfusion index − 0.176×((white blood cell count/100)⁻¹), where sex = 1 for male patients.

### Subgroup analysis by diagnosis

**Fig. 3:**
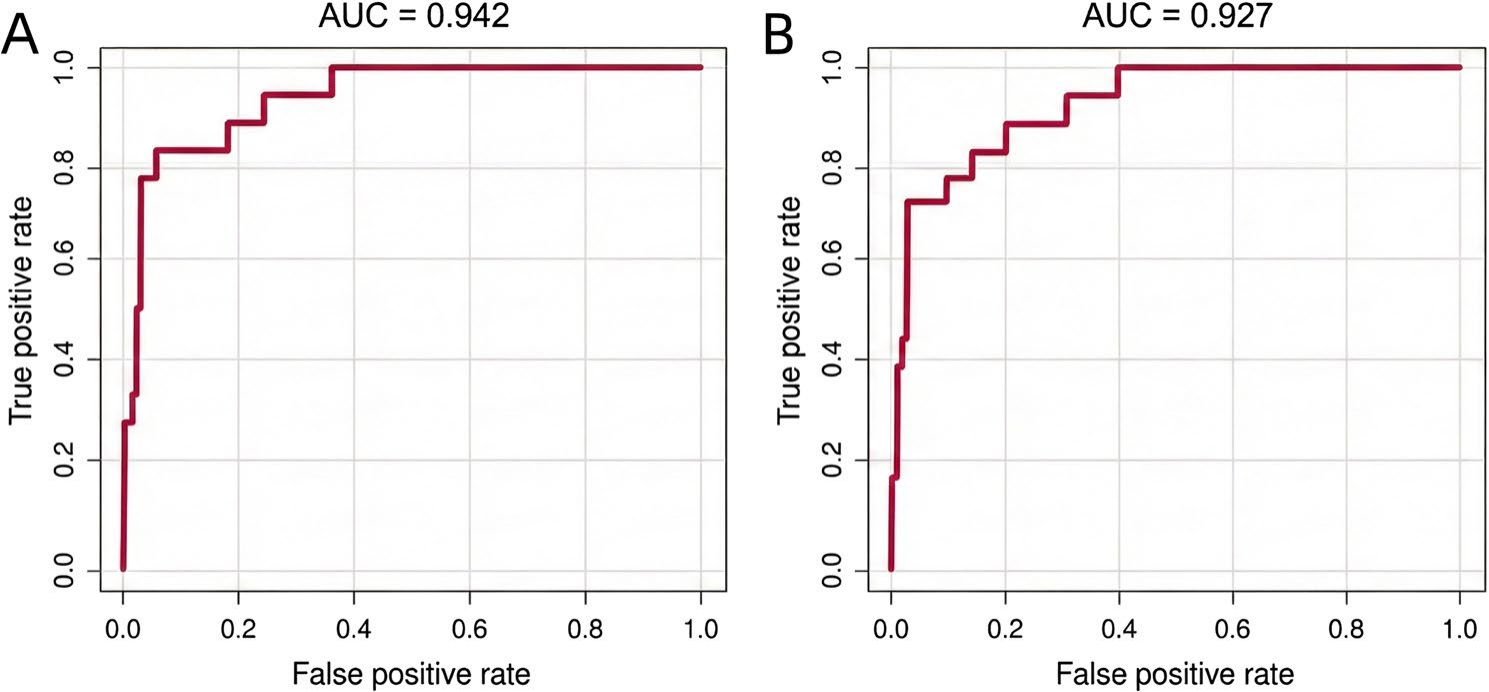
Early D-dimer/fibrinogen has high precision for predicting in-hospital mortality in the subgroups. Receiver operating characteristic curves for in-hospital mortality in the subgroups in the multivariable model. **a** The curve models the equation: In-hospital mortality = − 1.183 − 0.038×age + 0.369×central venous pressure + 0.478×DFR + 1.994×(sex = 1) − 0.047×hemoglobin + 0.033×lactate + 0.024×lymphocyte count + 1.989×neutrophil count − 1.204×PI − 0.026×((white blood cell count/100)⁻^2^), where sex = 1 for male patients. **b** The curve models the equation: In-hospital mortality = − 5.146 + 0.020×age − 0.495×central venous pressure + 0.455×DFR − 0.495×(sex = 1) + 0.007×hemoglobin − 0.115×lactate + 0.268×lymphocyte count + 0.310×neutrophil count + 1.431×(perfusion index^− 0.5^) + 0.094×white blood cell count, where sex = 1 for male patients. AUC, area under the curve; DFR, D-dimer/fibrinogen ratio

In the dissection/aneurysm subgroup, DFR was significantly associated with in-hospital mortality and increased NT-proBNP in the multivariable model (Table 4). In the Thrombotic diseases subgroup, DFR was significantly associated with shorter length of stay, fewer ICU-free days, and less mechanical ventilation-free time. No significant associations between DFR and other outcomes were observed in either subgroup (all *P* > 0.05). The receiver operating characteristic curves for in-hospital mortality in the dissection/aneurysm and thrombosis-related subgroups had AUCs of 0.942 and 0.927 (Fig. 3a and b), showing excellent predictive value.

**Table 4:** Predictive value of DFR for primary and secondary outcomes in the vascular disease sub-groups

	Dissection/Aneurysm	*P*-value	Thrombotic diseases	*P*-value
In-hospital mortality	1.61 (1.02, 2.54)	0.04	1.58 (0.90, 2.76)	> 0.10
Length of stay (days)	−2.21 (− 5.80, 1.38)	0.23	**−2.46 (− 4.43**,** − 0.49)**	**< 0.0** **5**
ICU-free days	−2.05 (− 5.29, 1.19)	0.22	**−1.76 (− 3.47**,** − 0.06)**	**< 0.05**
MV-free time (h)	−1.94 (− 5.39, 1.52)	0.27	**−2.22 (− 4.09**,** − 0.35)**	**< 0.05**
P/F	16.07 (− 9.86, 42.00)	0.23	−2.49 (− 30.48, 25.51)	> 0.10
MAP (mmHg)	−0.35 (− 1.38, 0.68)	0.66	0.33 (− 1.33, 2.00)	> 0.10
Platelets (×10^9^/L)	−5.97 (− 14.722, 0.78)	0.18	−4.33 (− 17.01, 8.34)	> 0.10
Total bilirubin (µmol/L)	−1.42 (− 4.16, 1.32)	0.31	−4.31 (− 11.68, 3.05)	> 0.10
Creatinine (µmol/L)	14.96 (− 7.13, 37.04)	0.19	1.83 (− 20.19, 23.84)	> 0.10
NT-proBNP (pg/mL)	**910 (81**,** 1**,**739)**	**0.03**	−72 (− 1,064, 921)	> 0.10

Model is adjusted for age, central venous pressure, sex, hemoglobin, lactate, lymphocyte count, neutrophil count, perfusion index, and white blood cell count

*MAP* Mean arterial pressure, *MV* Mechanical ventilation

Bold: The effect size is statistically significant

## Discussion

Our retrospective analyses revealed that early DFR in critically ill patients requiring vascular surgery had high predictive value for the primary outcome of in-hospital mortality, as well as the secondary outcomes of platelet count and NT-proBNP level. The high degree of accuracy and robustness of early DFR for pathologies spanning aortic dissection/aneurysm to Thrombotic diseases suggests that DFR may be an effective prognostic tool for patients requiring vascular surgery who are managed in the ICU.

The non-survivor group exhibited a shorter hospital stay and fewer ICU-free days in our cohort of ICU-admitted patients with vascular pathologies, as is commonly seen in critical care settings. This typically reflects an early, severe clinical trajectory rather than lower illness burden [21]. While a protracted stay in hospital is associated with poor outcomes, exceedingly short stays can be associated with early mortality [21]. Patients who die often do so early, before prolonged ICU support and step-down care become possible. In vascular diseases, early death can be driven by refractory shock, uncontrollable bleeding, massive thromboembolic obstruction, rapid progression of malperfusion, or perioperative catastrophic complications [22–24]. Therefore, shorter length of stay in non-survivors should be interpreted as an indirect sign of severity or death shortly after ICU admission.

Indeed, this is consistent with the accompanying physiologic and laboratory profiles in our cohort. Non-survivors had higher heart and respiratory rates and higher lactate levels, indicating a stronger systemic stress response and, plausibly, worse tissue perfusion. Lactate is a well-established marker of shock physiology and adverse outcomes across ICU populations, and its elevation in vascular emergencies is clinically consistent with malperfusion syndromes or cardiopulmonary collapse [25, 26]. In addition, non-survivors showed higher inflammatory indices (e.g., leukocytosis) and markers of organ injury. Taken together, the non-survivors appear to present a phenotype characteristic of global physiologic derangement, in which dysregulated coagulation and fibrinolysis—as reflected by elevated DFR—may be both a consequence of the underlying dysregulation and a contributor to downstream organ dysfunction through microvascular thrombosis and endothelial injury [11, 12].

Importantly, our results also highlight that DFR is not merely associated with in-hospital mortality, but is aligned with organ dysfunction signals. In the overall cohort, higher DFR tracked with lower platelet counts and higher cardiac stress biomarkers (i.e., NT-proBNP), and these relationships remained evident even after accounting for baseline and early physiologic covariates [4, 19, 27–31]. This coherence across systems supports the idea that DFR may serve as a “severity integrator” in vascular ICU patients, reflecting a systemic thrombo-inflammatory state that manifests simultaneously as consumptive thrombocytopenia, myocardial strain, and impaired organ perfusion [32].

We observed high predictive performance for DFR across a spectrum of vascular pathologies with distinct mechanistic bases. The mechanisms associated with aortic dissection/aneurysm include tissue-factor–mediated coagulation activation, platelet activation, and false-lumen thrombosis. These may evolve toward a consumption-dominant coagulopathy while also amplifying systemic inflammation, which can jointly drive high D-dimer and relative depletion of fibrinogen reserve; in this context, a high DFR is a plausible marker of severe disease and systemic derangement [12, 15, 17]. Aortic pathology also frequently intersects with major open/endovascular interventions and aggressive hemodynamic control. Perioperative hemodilution, transfusion, and targeted hemostatic therapies can modify fibrinogen and D-dimer values, so DFR in this setting may reflect the net effect of injury severity plus early resuscitation and procedural physiology [15–17]. Accordingly, within dissection/aneurysm phenotypes, DFR may align more closely with early catastrophic hemodynamic consequences and organ stress (e.g., tamponade, aortic regurgitation, myocardial ischemia, and malperfusion shock), making in-hospital mortality associations more prominent.

In thrombotic diseases, the mechanisms underlying the pathology are distinct. Fibrinogen may remain normal, rise as an acute-phase reactant, or be consumed, which is typical in massive thromboses or when systemic inflammation and multi-organ dysfunction coexist [15, 17]. Therefore, in thrombotic diagnoses, a higher DFR may still capture high fibrin turnover relative to fibrinogen reserve, but it may map more strongly onto illness complexity and support needs (ICU/ventilation course) than onto immediate lethality alone—particularly when prompt anticoagulation and reperfusion strategies can alter trajectories [4, 20].

These diagnosis-dependent patterns of DFR emphasize that the same value may carry different implications depending on the underlying vascular condition and its treatment pathway. DFR is therefore best interpreted within diagnosis-aware risk stratification rather than as a universal cutoff. This approach is consistent with current disease-specific prognostic efforts in vascular critical care; for example, risk scoring in type B aortic dissection has been studied to refine prognostic assessment beyond bedside impression, revealing that a novel composite tool that incorporates DFR among age, ejection factor, and other laboratory measures had superior predictive accuracy compared with other predictive tools [33, 34]. DFR may provide a complementary laboratory dimension in such disease-specific frameworks, especially at early ICU timepoints when imaging-derived severity and malperfusion status may not yet be fully characterized [33, 34].

Our modeling results suggest that DFR provides incremental prognostic information for in-hospital mortality beyond routinely available clinical and laboratory variables [14, 33, 35]. This represents an improvement over many common ICU triage markers—vital signs, lactate, leukocyte count, hemoglobin—which capture global physiologic stress but do not offer specific insights into the coagulation–fibrinolysis axis. Conceptually, DFR adds a hemostatic “layer” to early risk assessment by encoding fibrin turnover relative to fibrinogen reserve [15, 17]. In our study, the model incorporating DFR achieved good discrimination for in-hospital mortality, and subgroup performance suggested that DFR can contribute meaningfully to prognosis estimation when interpreted in a diagnosis-aware manner [13, 14, 35].

A key advantage is feasibility: DFR is computed from the results of standard laboratory tests that are available within the first 24 h, supporting early decisions [13, 14, 35, 36]. This is the period when vascular ICU clinicians must make high-stakes decisions—hemodynamic targets, timing of imaging reassessment, the need for invasive monitoring, anticoagulation strategy, and perioperative hemostatic planning [4, 19]. A marker that reflects the coagulation–fibrinolysis state early may therefore complement physiologic assessment and organ function scores, particularly in mixed vascular cohorts where the dominant problem may shift rapidly between bleeding risk, thrombosis risk, and malperfusion. Our findings support this practical role: higher DFR was consistently observed among patients with worse hospital outcomes, suggesting that DFR captures clinically meaningful severity information in this setting [9, 37].

Interpretability is another strength. Although multivariate models can be complex, DFR itself aligns with a coherent pathophysiological narrative. The association of higher DFR with lower platelet counts and higher cardiac stress biomarkers (e.g., NT-proBNP) in our cohort supports internal biological consistency rather than an isolated statistical artifact [29, 31, 32]. This suggests that DFR may help clinicians connect laboratory evidence of thrombo-inflammatory activation with concurrent organ stress, reinforcing its potential bedside utility [11, 12]. These features of DFR for risk stratification could improve patient management and support critical care resource allocation [4].

However, DFR should not be overextended as a universal predictor of all clinically relevant outcomes. In our overall cohort, DFR showed less robust adjusted associations with several utilization-type outcomes, including length of stay, ICU-free time, and mechanical ventilation-free time. These endpoints are influenced by non-biological factors (e.g., protocols, rehabilitation, bed availability, and extubation practices) and by competing risks (e.g., early death truncating time at risk). Thus, within this dataset, the most consistent role for DFR is as an early marker associated with in-hospital mortality and selected organ dysfunction signals. Its relationships with course indicators appear more context- and diagnosis-dependent.

Our findings should be interpreted within the context of the limitations of the study. First, the retrospective design inherently limits control over measurement timing, completeness, and granularity of variables. Some clinically important determinants—such as detailed malperfusion classification in dissection, imaging-derived clot burden in pulmonary embolism, right ventricular function metrics, timing from symptom onset to ICU admission, and precise treatment sequencing—may not be uniformly captured or may vary in documentation quality. As a result, residual confounding remains possible even with multivariate adjustment. Second, DFR was evaluated using early laboratory values, which provides immediate clinical utility but does not capture dynamic changes. Hemostatic biomarkers can change rapidly in response to intervention (e.g., surgery, thoracic endovascular aortic repair, anticoagulation, transfusion, or fibrinogen replacement) and disease progression. Serial trends may outperform single measurements for predicting both deterioration and recovery. However, standardizing serial sampling and aligning it with treatment timelines is difficult to accomplish retrospectively and would require prospective design features. Third, therapeutic actions can directly influence the two components of DFR, creating feedback between clinicians’ responses and measured biomarkers. For instance, transfusion strategies, fibrinogen supplementation, or anticoagulation adjustments may shift fibrinogen or D-dimer levels; conversely, high D-dimer or low fibrinogen levels may prompt clinicians to treat differently. This treatment–biomarker interplay is an inherent challenge for biomarker prognostication in real-world ICU care, and we did not adjust for the impact of clinical interventions, which represent potential confounding factors that must be addressed in future studies. Given the dynamic nature of DFR, we focused on preoperative measurement, prior to surgical intervention in the ICU. Further refinement of the optimal point in the treatment timeline for measurement will be necessary to develop DFR assessment as a prognostic tool for patients requiring vascular surgery in the ICU. Fourth, given the small number of deaths in the overall cohort, the results may be statistically overfitted. As such, the results warrant cautious interpretation and confirmation in future cohorts. Large prospective studies are needed to validate the generalizability of the findings and standardize the approach for prognostic DFR measurement in these patients. Moreover, as the model lacks an independent external validation cohort, future studies will be required to test the model on external datasets to confirm its generalizability.

## Conclusions

This study suggests that early DFR could be developed as a prognostic indicator for ICU-admitted vascular patients. Reflecting the complex interplay between severe coagulopathy and cardiac stress, DFR may serve as a biological sentinel for systemic deterioration. The DFR-based multivariate model demonstrated high predictive accuracy, highlighting its potential to support clinical decision making through early, precise risk stratification. By repurposing routine laboratory parameters into a prognostic tool, DFR offers a cost-effective strategy to optimize management and improve survival in this vulnerable population. Future research should prioritize large-scale, prospective multi-center studies to validate these findings and evaluate the potential of DFR-guided therapeutic interventions in clinical practice.

## Data Availability

All datasets used and analyzed during the current study are available from the corresponding author on reasonable request.

## Abbreviations

AUC: Area under the curve
bpm: beats per minute
DFR: D-dimer-to-fibrinogen ratio
DX: Diagnosis
CR: Creatinine
CVP: Central venous pressure
EMR: Electronic medical record
HGB: Hemoglobin
ICU: Intensive care unit
LOS: Length of stay
MAP: Mean arterial pressure
MV: mechanical ventilation
NT-proBNP: N-terminal pro-B-type natriuretic peptide
P/F: Ratio of arterial oxygen partial pressure to fractional inspired oxygen
PLT: Platelet count
ROC: Receiver operating characteristic
